# Two maize *END-1* orthologs, *BETL9* and *BETL9like*, are transcribed in a non-overlapping spatial pattern on the outer surface of the developing endosperm

**DOI:** 10.3389/fpls.2014.00180

**Published:** 2014-05-06

**Authors:** Joaquín Royo, Elisa Gómez, Olivier Sellam, Denise Gerentes, Wyatt Paul, Gregorio Hueros

**Affiliations:** ^1^Departamento Biomedicina y Biotecnología (Genética), Universidad de AlcaláMadrid, Spain; ^2^GM Trait Discovery, Biogemma – Centre de Recherche de ChappesChappes, France

**Keywords:** nsLTP, transfer cells, endosperm, aleurone, maize, *Arabidopsis*

## Abstract

In the course of a project aimed to isolate transfer cells-specific genes in maize endosperm we have identified the *BETL9* gene. *BETL9* encodes for a small protein very similar in sequence to the product of the barley transfer cell-specific gene *END-1*. Both BETL9 and END-1 proteins are lipid transfer proteins, but their function is currently unknown. *In situ* hybridization analysis confirms that the *BETL9* gene is exclusively transcribed in the basal endosperm transfer cell layer during seed development since 10 days after pollination. However, immunolocalization data indicates that the BETL9 protein accumulates in the maternal placento-chalaza cells located just beside the transfer cell layer. This suggests that the BETL9 protein should be transported to the maternal side to exert its, still unknown, function. In addition, we have identified a second maize gene very similar in sequence to *BETL9* and we have named it *BETL9like*. *In situ* hybridization shows that *BETL9like* is also specifically transcribed in the developing maize endosperm within the same time frame that *BETL9*, but in this case it is exclusively expressed in the aleurone cell layer. Consequently, the *BETL9* and *BETL9like* genes are transcribed in a non-overlapping pattern on the outer surface of the maize endosperm. The *BETL9* and *BETL9like* promoter sequences, fused to the GUS reporter gene, accurately reflected the expression pattern observed for the genes in maize. Finally, we have identified in the *Arabidopsis* genome a set of four genes orthologous to *BETL9* and *BETL9like* and analyzed the activity of their promoters in *Arabidopsis* transgenic plants carrying fusions of their promoter sequences to the GUS reporter. As in the case of the maize genes, the *Arabidopsis* orthologs showed highly complementary expression patterns.

## INTRODUCTION

Developing seeds are strong sinks for nutrients produced in the maternal plant. Since there are not symplastic connections between the maternal and filial tissues, the flow of nutrients required to sustain seed filling has to be carried through the apoplast, where solutes coming from the vascular system of the mother are discharged in the maternal side. In many species, the nutrients are uploaded from the apoplast by a specialized group of cells that differentiate as transfer cells in the outer surface of the developing seed, facing the maternal vascular terminals (reviewed in Royo et al., 2007). Transfer cells are characterized by numerous cell wall ingrowths that increase their surface area to enhance its nutrient uptake capacity (Pate and Gunning, 1972). In cereals, these transfer cells differentiate from the endosperm and their precise position and extension varies on the different species. In maize, the maternal vascular terminals are placed at the base of the developing seed and form a cup-shaped cushion. Nutrients discharged from the conducting vases must transverse several layers of crushed maternal cells, the placento-chalazal zone (Kladnik et al., 2004), in their way to the seed surface. There, the transfer cells are located as a continuous layer placed in front of the maternal vascular terminals and interrupting the aleurone layer that covers the remaining surface of the seed (Thompson et al., 2001). They constitute the basal endosperm transfer cell layer (BETL). In wheat and barley, there is a vascular bundle running along the length of the grain and the nutrients are discharged, through modified maternal cells in the nucellar projection, to the endosperm cavity that extends along the seed, in parallel to the vascular bundle. From this cavity, nutrients are uptaken by the endosperm transfer cells whereas the aleurone covers the remaining surface of the seed (Olsen, 1992; Royo et al., 2007). In rice, nutrients transported in the vascular bundle running along the length of the developing seed are not discharged to a cavity, but loaded symplastically into a layer of nucellar epidermal cells that covers most of the seed surface and it is of maternal origin. Therefore, nutrients from this nucellar tissue do not move to the apoplast facing the filial endosperm in a localized region and consequently there is no a distinct transfer cell layer differentiated from the aleurone (Bechtel and Pomeranz, 1977; Oparka and Gates, 1981; Krishnan and Dayanandan, 2003). Nevertheless, the region of the aleurone facing the vascular bundle contains additional layers of cells and these cells possess a specific regulatory program, as evidenced by the expression of genes not found in any other area of the aleurone (Li et al., 2008; Kuwano et al., 2011).

A growing number of genes expressed in the endosperm transfer cells have been identified during the last years. In maize there is already an extensive collection that includes genes named as *BETL1*, *2*, *3,* and *4* (Hueros et al., 1995, 1999) encoding for small cysteine-rich proteins. The product of the *BETL2* gene was renamed BAP-2 (basal layer antifungal protein 2) after its *in vitro* antifungal activity was demonstrated (Serna et al., 2001). *meg-1* (*maternally expressed gene1*) encodes for another small cysteine-rich protein that shows imprinting in their expression (Gutiérrez-Marcos et al., 2004). *ZmCKS* encodes for the enzyme CMP-KDO synthase involved in the activation of an important precursor for the synthesis of components of the cell wall pectins and, although not transfer cells specific, it is highly expressed in this tissue, probably to sustain the intensive remodeling of their cell walls during development (Royo et al., 2000). ZmMRP-1 is a single-MYB domain transcription factor of the SHAQKYF class that controls the expression of several of the *BETL* genes (Gómez et al., 2002) and *meg-1* (Gutiérrez-Marcos et al., 2004) and it is a major determinant of the identity of the endosperm transfer cells (Gómez et al., 2009). *ZmTCRR-1* and *ZmTCRR-2* encode Type A response regulators that are probably involved in a signal transduction pathway important for the development of these cells and both genes are also transcriptionally regulated by ZmMRP-1 (Muñiz et al., 2006, 2010).

In barley the *END-1* (*endosperm 1*) gene is expressed in the transfer cells facing the endosperm cavity (Doan et al., 1996) and it encodes for a non-specific lipid transfer protein (nsLTP). nsLTPs are a large family of proteins widely distributed across the plant kingdom that are able to bind lipids in a hydrophobic cavity stabilized by four disulphide bridges among a set of eight conserved cysteines in their primary sequence (reviewed in Yeats and Rose, 2008). Some of the nsLTPs genes are expressed in aleurone cells and could participate in the synthesis of extracellular cuticle layers (Boutrot et al., 2007), but a similar activity in transfer cells seems at odds with the active transport of nutrients circulating through these cells. Subsequently, it has been shown that other nsLTPs similar to END-1 are expressed in transfer cells from wheat (Kovalchuk et al., 2009, 2012) and in the maternal nucellar cells covering the vascular bundles and the adjacent aleurone cells that are functionally equivalent in rice (Li et al., 2008). An alternative role in antipathogen defense has be hypothesed for nsLTPs, consistently with the emerging evidence suggesting that endosperm transfer cells control a delicate balance between the need to facilitate an intense transport and the need to impede the ingress of pathogens into the growing seed.

In this paper we describe the cloning and characterization of *BETL9*, a maize gene for a nsLTP highly related to *END-1* and its wheat and rice relatives. *BETL9* was cloned in the course of a screening for *BETL* genes and *in situ* hybridization confirmed that it is really specifically expressed in that tissue. We have found that there is a highly similar gene to *BETL9* in maize, that we named *BETL9like*. Surprisingly, *BETL9like* is expressed instead in the aleurone cells, *in situ* hybridization with specific probes for both genes showed that there is a sharp boundary between their expression domains that coincides with the border between the BETL and aleurone regions of the endosperm. We have isolated proximal promoter regions for both genes that reproduce, in transgenic maize plants, the expression pattern of the endogenous genes. We have also isolated the promoter regions of the four *Arabidopsis* genes encoding for the nsLTPs most closely related to *BETL9* and *BETL9like* and studied their expression patterns in transgenic *Arabidopsis* plants carrying promoter-GUS constructs.

## RESULTS

### ISOLATION OF TWO LIPID TRANSFER GENES SPECIFICALLY EXPRESSED IN THE DEVELOPING MAIZE KERNEL

**FIGURE 1:**
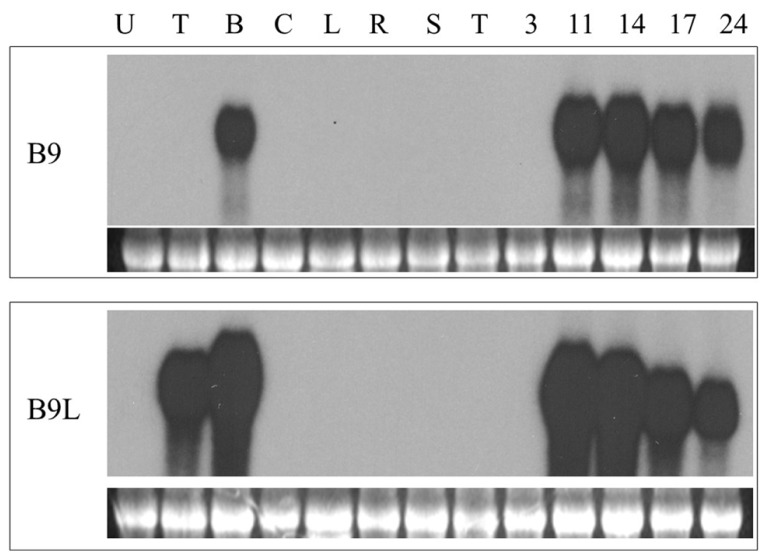
**Northern blot analyses of the expression of *BETL9* and *BETL9like*.** The probe used in each northern blot is indicated on the left. Each panel contain an EtBR picture of the ribosomal RNA to show equal loading. The tissues used as source of total RNA (10 μg per lane) were: U, unpollinated female flowers; T, upper part of 10 DAP kernels; B, lower part of 10 Dap kernels; C, coleoptiles; L, leaves; R, roots; S, silks; T, tassel (male flowers); 3, 11, 14, 17, and 24, complete kernels collected at the indicated developmental stage (in DAP).

We have previously described (Balandin et al., 2005) a screening for genes specifically expressed in the basal halves of developing maize kernels, based on the method of Sokolov and Prockop (1994). In the course of this screening we isolated a cDNA clone containing an open reading frame for a 107 amino acids long nsLTP. Northern blot analysis indicated that the transcripts corresponding to this cDNA were only detectable in developing maize kernels as early as 11 days after pollination (DAP; the youngest time point used in these experiments) and only in RNA extracted from the lower halves of hand dissected kernels (**Figure 1**). In accordance with its expression pattern we have called this gene *BETL9*. Inspection of the Maize Genome Sequencing Project database reveals that this gene is located on chromosome 3 and has the identifier GRMZM2G0847413.

Analysis of the maize genome sequence database revealed the presence of a gene closely related to *BETL9* that we consequently named as *BETL9like*. A cDNA clone for *BETL9like* was isolated and Northern blot analysis indicated it was also specifically expressed in developing maize kernels, with a temporal pattern closely resembling that of *BETL9*. Its transcripts, however, were not restricted to the basal halves of the kernels, the RNA from the upper halves of immature kernels showed an equally strong hybridization signal with the *BETL9like* probe (**Figure 1**). According to the Maize Genome Sequencing Project database this gene is located on chromosome 8 and has the identifier GRMZM2G091054.

Non-specific lipid transfer protein are an extensive group of related proteins widely distributed in the plant kingdom. Plant nsLTPs typically contain an N-terminal signal peptide and are characterized by an eight cysteine motif (8 CM) of the form: C-Xn-C-Xn-CC-Xn-CXC-Xn-C-Xn-C. The cysteine residues are engaged in the formation of four disulphide bonds that stabilize a hydrophobic cavity where phospholipids and other lipidic compounds can bind (Yeats and Rose, 2008). Consequently, the *in vivo* activity of nsLTPs is supposed to pivot on this ability to bind lipids. However, the actual physiological function of most of these proteins has not been determined. Based on sequence relationships among their rice, wheat and *Arabidopsis* members, plant ns-LTPs have been previously classified in nine groups (Boutrot et al., 2008; Wang et al., 2012) and according to this scheme *BETL9* and *BETL9like* belong to group VI. Edstam et al. (2011) classified the plant nsLTPs proteins using a different sorting scheme and included in their analysis sequences from non-flowering plants. In their scheme, nsLTPs of group VI are included in a wider type they named D. Type D nsLTPs appeared very early in evolution, shortly after the divergence of the first land plants.

**FIGURE 2:**
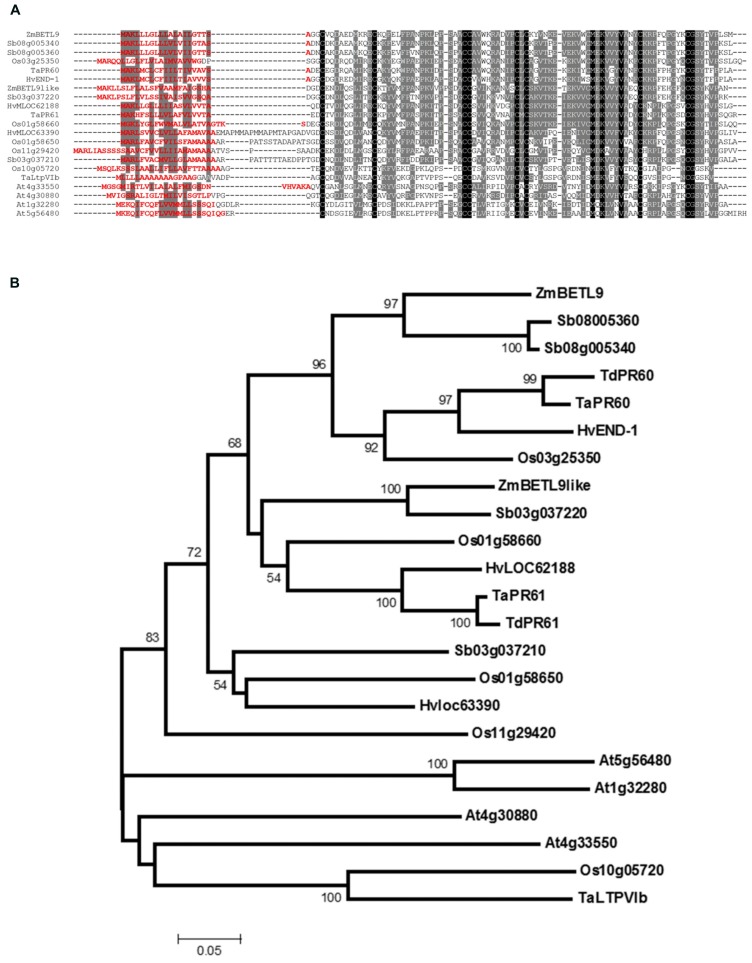
**Sequence analysis of the protein sequences of group VI nsLTPs. (A)** Alignment of type VI nsLTPs proteins from maize (ZmBETL9 and ZmBETL9like), sorghum (Sb03g037210, Sb03g037220, Sb08g005340, and Sb08005360), barley (HvEND-1, HvMLOC62188, and HvMLOC63390), rice (Os01g58650, Os01g58660, Os03g25350, Os10g05720, and Os11g29420), wheat (TaLtpVIb, TaPR60, and TaPR61), and *Arabidopsis* (At1g32280, At4g30880, At4g33550, and At5g56480). Sequences were aligned using ClustalW. Identical amino acids are in black boxes, similar amino acids in gray boxes. Signal peptides were predicted using SignalP 4.1 (Peterson et al., 2011) and their sequences are shown in red. **(B)** Phylogenetic relationships of the nsLTPs protein from type VI. The analysis is based on the alignment of the proteins shown in **A**, excluding the N-terminal signal peptides, plus the *Triticum durum* TdPR60 and TdPR61 proteins. The Neighbor-Joining tree was constructed using MEGA 5.2 with 1000 bootstrap replicates and the *p*-distance and pairwise deletion options. Only bootstrap values above 50% are shown.

Based on the Boutrot et al. (2008) and Wang et al. (2012) studies and our own search on sequence databases we have compiled the complete amino acid sequences of members of the group VI from several cereal species, along with the four members from *Arabidopsis* (**Figure 2A**). In the alignment shown in **Figure 2A** we include the two maize proteins BETL9 and BETL9like, but not the translation product of the AC194405.3_FG009 gene. This gene, located on chromosome 8, contains the N-terminal half of a related protein, but its coding region is currently interrupted in the sequence databases by a region of poor sequence quality that makes impossible its precise identification. In **Figure 2A** we have also included the sequences of the proteins encoded by three classes of wheat genes: *TaLtpVIb* from *Triticum aestivum* (Boutrot et al., 2008) and the *T. aestivum* members of the two pairs of orthologous *T. aestivum* and *T. durum* genes *TaPR60* and *TdPR60* (Kovalchuk et al., 2009) and *TaPR61* and *TdPR61* (Kovalchuk et al., 2012). *TaPR61* corresponds to the wheat gene *TaLtpVI.a* described by Boutrot et al. (2008). We also include the four rice genes (Os01g58650, Os01g58660, Os10g05720, and Os11g29420) initially reported by Boutrot et al. (2008), and Os03g25350, added by Wang et al. (2012). Os01g58660 was cloned on the basis of its sequence similarity with *TaPR60* by Li et al. (2008), and named *OsPR602*. From barley we have included the product of *END-1* (Doan et al., 1996), located on chromosome 1H, and those from the genes HvMLOC62188, on chromosome 3HL, and HvMLOC63390, on chromosome 3H. Finally, we include four genes from *Sorghum bicolor*, two of them (Sb08g005340 and Sb08g005360) probably represent the product of a recent tandem duplication event, since they are highly similar and located in close proximity on chromosome 8. The same duplication event could have generated the nearby and very similar gene Sb08005345, but we do not include it here because it contains sequence alterations suggesting this might be a pseudogen. The other pair of sorghum genes (Sb03g37210 and Sb03g37220) are located in close proximity on chromosome 3, but their sequences are not as closely related as that of the genes on chromosome 8. Wang et al. (2012) included in group VI two additional genes from rice (Os11g03870) and *Arabidopsis* (At2g13295), but we discarded them here because their protein sequences do not have all the characteristic features of the members of group VI. These features are the presence of a valine and methionine residues located at positions -4 and -10, respectively, from cysteine at position 7 of the motif 8 CM, and a single intron interrupting their coding sequence 4 nt downstream of the codon for the last cysteine of the 8 CM motif.

Phylogenetic analysis based on the alignment of the sequences of the mature nsLTPs reveals the presence of two subgroups of proteins of cereal origin in type VI (**Figure 2B**). The first one includes BETL9 and the product of the barley gene *END-1* (Doan et al., 1996). *END-1* is expressed in the endosperm transfer cells that in the barley developing kernel are located over the nucellar projection running along the length of the grain. This subgroup also includes the products of the common and durum wheat endosperm transfer cell-specific genes *TaPR60* and *TdPR60* (Kovalchuk et al., 2009) as well as the protein from the rice gene Os03g25350 and from the two closely related sorghum genes located in close physical proximity on chromosome 8. Microarray database mining reveals that the Os03g25350 transcripts are abundant in spikelets and young seeds, but we do not have precise data about the pattern of expression of this gene. The second subgroup includes BETL9like, and the protein products of the genes HvMLOC62188 from barley, located on chromosome 3HL, Sb03g037220 from sorghum, Os01g58660 from rice and *TaPR61,* and *TdPR61* from common and durum wheat. The wheat genes are expressed in the endosperm transfer cells, but also in the aleurone, part of the starchy endosperm, the embryo surrounding region (ESR) and the developing embryo (Kovalchuk et al., 2012). Os01g58660 is expressed in the aleurone cells located in most close proximity to the maternal vascular bundles, and in the maternal tissues located between those vascular bundles and the seed surface (Li et al., 2008). The remaining proteins of cereal origin of this group VI do not form a well-supported clade and there is not much information about the expression patterns of their genes.

The protein products from the four *Arabidopsis* genes belonging to group VI (At1g32280, At4g30880, At4g33550, and At5g56480) do not cluster with the cereal ones. This separation between the proteins from *Arabidopsis* and cereal species inside a group is very common in the nsLTPs family and precludes identifying orthology relationships among them (Boutrot et al., 2008).

### *IN SITU* LOCALISATION OF THE *BETL9* AND *BETL9LIKE* TRANSCRIPTS IN THE DEVELOPING MAIZE KERNEL

**FIGURE 3:**
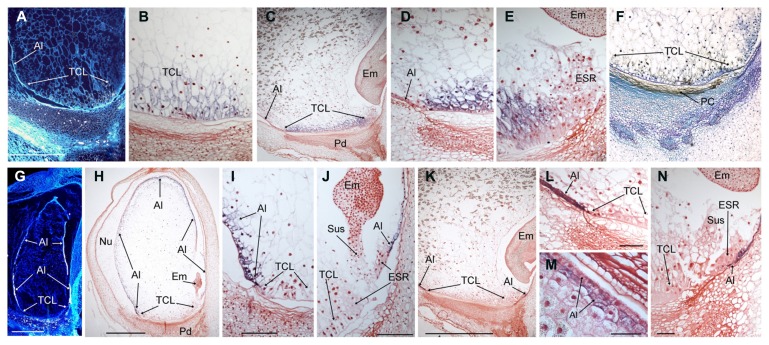
**Expression analyses of the *BETL9* and *BETL9like* genes in developing maize kernels.** The expression of *BETL9* was analyzed by *in situ* hybridization with antisense RNA probes **(A–E)** or Immunolocalization **(F)** with a specific antisera. The expression of *BETL9like* was analyzed with an antisense RNA probe (G to N). RNA labeling was done with S^35^-dUTP **(A,G)** or DIG-dUTP **(B–E,H–N)**. Sections correspond to 5 DAP **(A,G)**, 10 DAP **(B,F,H–J)**, and 16 DAP **(C–E,K–N)**. S^35^ hybridization signal appears as light dots in the dark field microphotographs, DIG signal is visualized as a purple–black precipitate. For the immunolocalization positive signal s visualized as a brown–black precipitate. Sense and preimmune sera controls produced no signal and are not shown due to space constrains. TCL, transfer cell layer; Al, aleurone; Em, embryo; ESR, embryo surrounding region; Pd, pedicel; PC, Placento-chalaza; Nu, nucella; Sus, suspensor. Bars mean 500 μm in **A,G**; 1 mm in **H,K** and 50 μm in **I,J,L–N**.

*In situ* hybridization experiments were used to clarify the spatial discrepancies observed in the Northern blot analyses, see above, where *BETL9* was localized in the lower half of the kernels whereas *BETL9like* was found both in the upper and lower halves. Localisation of the *BETL9* and *BETL9like* transcripts was determined in longitudinal sections of developing maize kernels using antisense probes corresponding to gene-specific regions of the cDNAs. *BETL9* transcripts are only present at the BETL (**Figures 3A–E**, TCL) whereas *BETL9like* transcripts are only at the aleurone cell layer covering the remaining outer surface of the developing endosperm (**Figures 3G–N**, Al). Both genes were readily detected as early as 5 DAP (**Figures 3A,G**). The transition between the transcription domains of both genes accurately reflects the transition between the characteristically elongated, cell wall ingrowths-covered transfer cells and the smaller, cubic, and symmetric aleurone cells. At the embryo pole, the aleurone consists of a single cell layer that forms a cavity enclosing the embryo, this layer is readily evidenced by the strong expression of the *BETL9like* marker. At the upper part of the seed (**Figure 3M**), the *BETL9like* marker seems to reflect the maturation stage of the aleurone cells, which at 16 DAP are still forming a three layers of cells that will evolve into a single cell layer at maturity. The hybridization signals appears in one, two, or three cell layers depending on the seed area analyzed. No transcripts were detected in the embryo, ESR or other parts of the seed and adjacent maternal tissues.

### LOCALISATION OF THE BETL9 PROTEIN

An antibody was raised in rabbits against the BETL9 mature protein translated in *Escherichia coli*. The antibody specifically recognized a single protein of the expected molecular size for the mature BETL9 protein, 9.8 KDa, in crude protein extracts prepared from the basal halves of 14 DAP maize kernels (data not shown).

Inmunolocalization using this antibody against wax-embedded sections from 14 DAP kernels detected the BETL9 protein exclusively at the placento-chalazal region located on the maternal tissues of the pedicel (**Figure 3F**). This maternal region is facing the BETL, where *in situ* studies indicate the *BETL9* transcripts are accumulating. Almost no signal is detectable at the BETL cells suggesting that the BETL9 protein is efficiently exported from them into the apoplast in a polarized fashion.

Assuming that the *BETL9like* transcripts are efficiently translated, the absence of inmmunolocalization signal either at the aleurone or the tissues surrounding it, suggests that this antibody is indeed specific against the BETL9 protein and does not appreciably detect BETL9like protein molecules.

### ACTIVITY OF THE *BETL9* AND *BETL9LIKE* PROMOTERS IN TRANSGENIC MAIZE

The promoter regions located upstream of the *BETL9* (1911 base pairs, bp) and *BETL9like* (2229 bp) coding regions were isolated from the corresponding clones of a BAC library screened with the cDNA probes. These promoter regions were fused to the reporter GUS gene and used to produce stable transformed maize transgenic plants. At least three transgenic lines containing single locus insertion of the constructs were analyzed in detail in each case.

**FIGURE 4:**
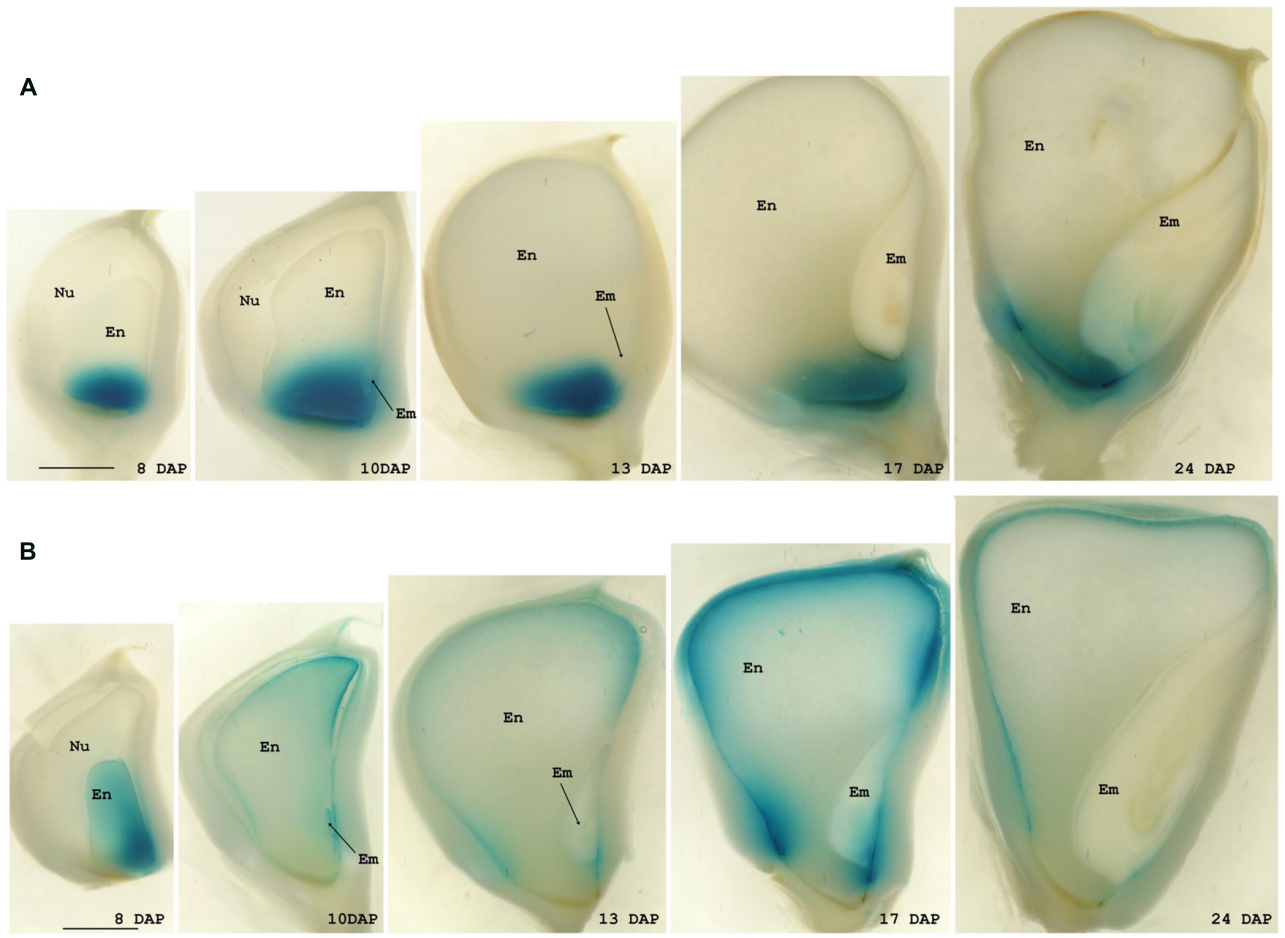
**Expression analyses of the *BETL9* and *BETL9like* promoters.** Histochemical analyses for GUS expression in maize kernels. Positive expression is seen as a blue precipitated. The age of the kernels is indicated as days after pollination. The promoters assayed are the *BETL9 promoter* **(A)** and the *BETL9like promoter* **(B)**, in the lower panel. En, endosperm; Em, embryo; Nu, nucella. The scale bars shown in the 8 DAP figures mean approximately 1 mm.

In the maize transgenic plants the pattern of GUS staining reproduced that obtained from *in situ* studies for both genes. The GUS signal was exclusively observed in developing seeds from 8 DAP until almost maturity in both cases but whereas in plants carrying the *BETL9* promoter the signal was only detected in the basal endosperm transfer cells (**Figure 4A**), in those with the *BETL9like* promoter the GUS staining was limited to the aleurone layer (**Figure 4B**). Consequently, these promoter regions are sufficient to explain the different expression patterns of both genes and post transcriptional processes are expected to be of minor importance in controlling it. The expression of the reporter constructs, as detected by histochemical staining, showed in both cases some delay when compared with the expression of the endogenous genes, although the difference in sensitivity between the GUS staining and the *in situ* hybridization might explain this discrepancy.

### ACTIVITY OF THE PROMOTERS OF THE *Arabidopsis BETL9*-RELATED GENES IN TRANSGENIC *Arabidopsis* PLANTS

**FIGURE 5:**
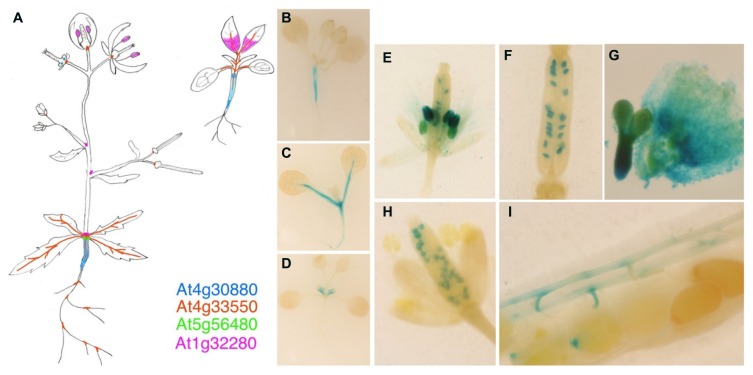
**Expression analyses of the *BETL9 A. thaliana* orthologs.** Histochemical analyses for GUS expression in *Arabidopsis thaliana* transgenic plants. **(A)** Cartoon showing the expression pattern registered for the four promoters assayed, as indicated by the color code. The signal inside the ovaries and immature siliques corresponds to At5g56480 and At1g32280. **B–D** are young plantlets (7–10 days after germination) transgenic for the promoters of At4g30880 **(B)**, At4g33550 **(C)**, and At1g32280 **(D)**. **(E–G)** Expression of At1g32280 in anthers and ovules **(E)**, immature seeds **(F)**, and embryo axis and endosperm **(G)**. **(H,I)** Expression of At5g56480 in immature seeds **(H)** and funiculus and micropilar endosperm **(I)**.

We have previously indicated that there are four *Arabidopsis* genes included in the group VI of nsLTPs (At1g32280, At4g30880, At4g33550, and At5g56480), but they do not cluster into either the *BETL9* or *BETL9like* subgroups. In the absence of clear phylogenetic relationships coming from the comparison of their sequences, analysis of their expression patterns could provide some insights into possible functional relationships between the *Arabidopsis* and cereal proteins. Consequently, we have isolated the promoter regions of the four genes by PCR amplification using forward and reverse primers designed from the sequence data at the *Arabidopsis* genomic sequence database. The resulting promoter regions were fused to the reporter GUS genes and used to obtain stable transgenic *Arabidopsis* plants. For each gene several transgenic lines were analyzed and the results of the more representative ones are shown (**Figure 5A**; for a color-coded cartoon). The promoters of the four genes show distinct patterns of activity with a very limited overlapping. In mature plants the activity of the promoter of At1g32280 is restricted to a collar region in the transition zone between the stem and hypocotyl, the basal rosette node, and in the stipules around the branching points of the inflorescence stems. In young seedlings this promoter is, however, transiently active in the first developing pair of true leaves, where it marks only the proximal parts of the leave (**Figure 5D**). At5g56480 is also active in the rosette node, but not in the stipules and neither in any part of developing plantlets. The At4g30880 promoter is active in the hypocotyl both in seedlings (**Figure 5B**) and mature plants. The expression of the gene is especially strong at the borders of the hypocotyl, i.e., at the interfaces with stem or roots. There is also a weak activity in nectaries. The activity of the At4g33550 promoter extends along the vascular bundles of stems, leaves, and root in the mature plant and developing plantlets (**Figure 5C**), and it is also evident in the collar of vascular bundles located at the base of flowers and immature siliques, as well as in the branching points of the root.

In reproductive tissues, the promoter of At1g32280 is active in mature anthers, ovules, and developing seeds (both in embryo and endosperm, **Figures 5E–G**) whereas that of At4g30880 is only transiently active in the developing anthers of young flower buds. The promoter of At5g56480 is active in ovules, from the bud to the mature stages, in the funicular connections to the pod vasculature and in very young seeds at the micropilar side (**Figures 5H,I**) but it is not active in developing seeds.

## DISCUSSION

The most characteristic feature of the nsLTPs is their well proved capacity to bind lipidic compounds (Yeats and Rose, 2008) and, consequently, it is widely assumed that they are probably involved in the transport of those substances. On the other hand, nsLTPs have a conspicuous N-terminal signal peptide that presumably leads to their export out of the cell and makes rather unlikely their participation in the intracellular trafficking of lipids. For this reason, it has been suggested that nsLTPs would be involved in the transport of lipids to extracellular locations during the synthesis of structures such as the cuticle layer (Pyee et al., 1994; Lee et al., 2009). This role would be particularly likely for those nsLTPs genes predominantly expressed in epidermal cells of aerial parts of the plant because it is there where the cuticular layers are usually located. However, it should be reminded that in each plant species there is a great number of nsLTPs that presumably have diversified to perform distinct physiological functions, and to work in different parts of the plant body and during diverse developmental stages. Consequently, it would be very surprising to find a single nsLTPs *in vivo* function. Indeed, some nsLTPs have shown antimicrobial activity *in vitro* and the expression of their encoding genes is induced after pathogen infection, suggesting that they could be involved in defense against these invaders (Molina and García-Olmedo, 1993, 1997; Guiderdoni et al., 2002; Gomès et al., 2003). For those nsLTPs their defensive role would be acting like toxic weapons, but for the *Arabidopsis* DIR1 (defective in induced resistance1) nsLTP it would be carrying, or chaperoning, a still unknown systemic signal that triggers defensive responses in parts of the plant still free of the pathogen (Maldonado et al., 2002; Champigny et al., 2013). It is not known if other nsLTPs could also be involved in the transport of lipid signals, in defense or developmentally related signaling pathways. On the other hand, some nsLTPs from legume plants participate in the control of the symbiotic interactions between the host and its rhizobium partner during the nodulation process, likely acting as a positive signal for the rhizobium colonization (Pii et al., 2009), but their exact mechanism of action is unclear.

In this context, *BETL9* and *BETL9like* are an interesting case because they are a couple of very similar maize genes exclusively transcribed in developing seeds, more specifically at the two endosperm layers covering their surface: *BETL9* in the *BETL* and *BETL9like* in the aleurone. Both cell layers can be considered as the endosperm epidermis, marking the border between the seed and the enclosing maternal body. However, the BETL and aleurone have very different physiological activities in that border because whereas the aleurone layer functions as a classical barrier, the BETL is a place of very active nutrient uptake into the developing seed from the maternal vascular terminals located just in front of it. In fact, the BETL is the only exchange surface available for the developing maize seed because the rest of the endosperm is surrounded by a cuticle layer (Davis et al., 1990). Consequently, we suggest that this pair of similar genes could have diversified following an ancient duplication to carry out different functions in those two contiguous regions. *BETL9like* in the synthesis of a cuticle, facilitating the establishment of an effective barrier surrounding the aleurone layer, and *BETL9* adopting a function more related to toxicity against pathogens trying to enter the seed along the same pathway that nutrients. That being the case, BETL9 would add to the number of proteins expressed at the BETL with presumed or already proven anti pathogenic activity. *BETL1*, *BETL3,* and *meg-1* (Hueros et al., 1995, 1999; Gutiérrez-Marcos et al., 2004) genes code for proteins related to the antimicrobial peptides defensins. BETL4 has some similarity to alpha-amylase/trypsin inhibitors (Thompson et al., 2001) and BETL2 was renamed as BAP-2 (basal layer antifungal protein 2) after been demonstrated that it has antifungal activity *in vitro* (Serna et al., 2001). In addition, both BETL1 and BAP2 proteins accumulate at the placento-chalazal area, the maternal region that the nutrients coming from the maternal vascular terminals, and presumably also the pathogens, should cross before arriving to the BETL. In the case of BETL9, the availability of an antibody allowed us to show that also in this case the protein is exported from the BETL to the apoplast and extends through the placenta-chalazal region where its putative antipathogenic activity should be exerted (**Figure 3F**). Either this export is particularly efficient, or the BETL9 protein molecules inside the BETL cells are not very stable because only traces of BETL9 protein are detected inside the BETL (**Figure 3F**). Unfortunately, we do not have an antibody able to detect the BETL9like protein and we cannot discern if it remains around the cell walls of the aleurone cells where it is transcribed, as expected if it is involved in the synthesis of the cuticle covering them, or it expands across the maternal cells as BETL9. Another, more speculative, possibility would be that BETL9 functions as a member of a signaling system between the seed and maternal tissues contributing to the coordinated development and location of the maternal vascular terminals and the BETL seed layer. We have already indicated that one nsLTP, the product of the *dir1 Arabidopsis* gene, is indeed involved in a signaling system. DIR1 protein belongs to group IV of nsLTPs according to the Boutrot et al. (2008) scheme, but groups IV and VI proteins were included in the wider Type D according to the classification scheme by Edstam et al. (2011), pointing to a relationship that could extend to their activities.

Independently of their putative physiological role, the expression patterns of *BETL9* and *BETL9like* genes provide us with a valuable material to study the mechanisms regulating differential gene activity between endosperm transfer cells and aleurone in maize. We have isolated proximal promoter regions of both genes that very closely recapitulate the expression patterns of the corresponding endogenous genes, in transgenic maize plants carrying promoter-GUS constructs (**Figure 4**). Consequently, these constructs could be used in the future to investigate the regulatory mechanisms determining BETL and aleurone specific expression. Unfortunately, there is still a very sparse knowledge about the transcription factors network regulating gene expression in the different maize endosperm domains. Conversely, the resulting scarcity of well characterized target sequences and their frequent ambiguity makes very difficult to get fruitful results only from sequence analysis. For this reason, we have attempted to complete the classical search for already characterized target motifs with a comparative approach in the sequence analysis of these promoters, taking advantage of the availability of genomic data from sorghum. Sorghum is a closely related species to maize and we reason that the three sorghum genes that we have identified as orthologous to either *BETL9* or *BETL9like* (Sb08g005340 and Sb08g005360 for *BETL9* and Sb03g037220 for *BETL9like*; **Figure 2B**) would presumably have similar expression patterns to their maize counterparts and be regulated by related transcription factors, binding similar target sequences. A search for conserved short motifs in the sequences of 1 Kb of the maize and sorghum proximal promoters of the five genes using the MEME (Bailey and Elkan, 1994) and MAST (Bailey and Gribskov, 1998) tools reveals that *BETL9* and Sb08g005360 have a significant number of common sequence motifs. *BETL9like* and Sb03g037220 share a different group of motifs, as expected from the distinct expression patterns of the maize genes and their relationships with their sorghum relatives. Intriguingly, although the protein products of the Sb08g005340 and Sb08g005360 genes are almost identical, the promoter of Sb08g005340 has a different pattern of motifs. Since both genes are closely located in the same chromosome, this suggests they could have differed in their expression patterns after a recent duplication event that generated them, although only experimental data could validate this prediction. Meanwhile, we have taken advantage of our conserved motifs approach to focus our attention on the presence in the promoter of the *BETL9* and Sb08g005360 genes of several copies of the sequence TATCT, sometimes repeated in tandem, and positioned around 500 bp upstream of the start codon of the coding region. This conserved motif is highly related, but not identical, to the “transfer cell box,” the target of the transfer cell specific transcription factor ZmMRP-1 (Barrero et al., 2006). In the promoters of *BETL9like* and Sb03g037220 there are several copies of the motif TTGACACTTG located between 200 and 400 bp upstream of the position of the start codon, this motif is also present in the promoters of the wheat *TdPR61* and barley HvLOC62188 genes that belong to the same phylogenetic group (**Figure 2B**). The first half of this motif resembles the W box-like sequences that are closely related to the binding motifs for WRKY proteins, although functional studies indicate they are unlikely targets for these transcription factors (Ciolkowski et al., 2008). The second half is related to the CANNTG E-box element found in other seed-specific promoters (Stalberg et al., 1996). Finally, we have found in the promoters of both *BETL9* and *BETL9like* genes and their sorghum relatives, but not in Sb08g005340, the motif ACATGCAAC, related to the RY repeat present in the promoters of several seed specific genes in cereals and legumes (Baumlein et al., 1992). This set of differentially conserved motifs comprises promising targets to be validated during the experimental dissection of both promoters.

The phylogenetic relationships among the cereal nsLTP proteins belonging to group VI suggests that a duplication before the divergence of the maize, sorghum, barley, rice, and wheat lineages originated the *BETL9* and *BETL9like* clades leaving apart a less well defined group of sequences (**Figure 2B**). Current data (Doan et al., 1996; Li et al., 2008; Kovalchuk et al., 2009, 2012; this work) indicates that this split has been accompanied by a differentiation in their expression patterns. The members of both main clades are specifically expressed in seeds, but with some differences. The genes of the *BETL9* clade from maize (**Figure 3**), the barley *END-1* (Doan et al., 1996) and the wheat *TdPR60* (Kovalchuk et al., 2009) have their expression restricted to the endosperm transfer cells. Endosperm transfer cells, although located in different physical arrangements in these species (Royo et al., 2007), share their position as entrance gates to the developing seed for nutrients coming from the maternal tissues. We do not have detailed data concerning the expression pattern of the rice member of this clade (Os03g25350), but the available information from microarray analysis is not incompatible with this scheme. On the other hand, the situation is not so homogenous in the *BETL9like* clade. The maize *BETL9like* gene is only expressed in the aleurone layer of the endosperm, and it is clearly silent in the transfer cells (**Figures 3** and **4**), but this clear-cut difference with the expression of its *BETL9* counterpart is not seen in the rice and wheat members of the clade. In rice there is not a distinct endosperm transfer cell layer, but the *OsPR602* (Os01g58660) expression is restricted to the aleurone cells located in closer proximity to the maternal vascular bundles that constitute their nearest equivalent (Li et al., 2008). Considering only this, the expression of *OsPR602* would be equivalent to that of the *BETL9* genes and not to the maize *BETL9like*. However, the promoter of *OsPR602* is also active in the maternal nucellar cells located in the pathway between the vascular bundles and the adjacent aleurone. This pattern contrasts with the expression of the typical rice transfer cell-like specific gene *AL1* (Kuwano et al., 2011) and indicates that *OsPR602* is not as specific as the *BETL9* genes. In addition, the wheat gene *TdPR61* (Kovalchuk et al., 2012) has also a wide expression pattern, including not only the aleurone, like the maize *BETL9like* gene, but the endosperm transfer cells, the ESR and the embryo, but not any maternal tissues surrounding the developing seed as in the case of *OsPR602*. Finally, the only source of information on the expression of the cereal genes of the group VI that cannot be clearly assigned to the *BETL9* or *BETL9like* clades comes from microarray experiments data for the three rice genes, and suggests that their transcripts are not restricted to developing seeds. On the absence of more precise data this could indicate that these genes have evolved to perform different physiological functions.

In conclusion, our current data indicates that in cereals there was a duplication and diversification of the genes encoding for the group VI nsLTPs leading to two clear subgroups, we have called them *BETL9* and *BETL9like* clades, that are mainly expressed in developing seeds, and a third one less well defined and studied. In maize the genes belonging to each of the two main subgroups have clearly different patterns of expression in the two endosperm domains covering the surface of the seed whereas in the other cereal species this spatial differentiation of the expression patterns is less clear. Aside from the cereal splits there are the four *Arabidopsis* genes belonging to group VI. As it is common in the other nsLTPs groups (Boutrot et al., 2008), the *Arabidopsis* proteins constitute a separate clade and it is not possible to establish an obvious correspondence between them and those from cereals. This suggests that the successive duplications leading to the current members in both groups of plant occurred after the divergence between the lineages leading to *Arabidopsis* and cereals. Data concerning the promoter activity of the four *Arabidopsis* genes (**Figure 5**) also support this notion because none of them have patterns reminiscent of those found for the cereal genes. The *Arabidopsis* genes have clearly diversified their spatial and temporal patterns of expression, suggesting they have evolved different *in vivo* functions, in addition their expression is not restricted to seeds although some members are expressed in immature endosperm and embryo.

## MATERIALS AND METHODS

### PLANT MATERIAL

DNA, RNA, and biological samples were obtained from wild type (var. A69Y) or transgenic (var. A188) maize plants (*Zea mays*) grown in a glasshouse and from *Arabidopsis thaliana* var Col-0 grown under long-day conditions (18 h light/6 h darkness, 70% humidity, 21°C/18°C).

### MOLECULAR BIOLOGY METHODS

Standard DNA and RNA methods were carried following Sambrock et al. (1989) and as previously described (Hueros et al., 1995).

### BACTERIAL EXPRESSION, PURIFICATION OF THE BETL9 PROTEIN AND PREPARATION OF THE ANTISERUM

The mature *BETL9* coding sequence was generated by PCR from the cDNA clone by PCR, using primers which added suitable restriction sites. The PCR product was digested with the restriction enzymes Bam HI and BglII and cloned into the pQE60 expression vector (Qiagen GmbH) to yield pQE60-BETL9. The pQE60 vector provides a start codon followed by three aminoacids, which results in the addition at the N-terminus of four aminoacids (MGGS) to the first residue of the mature BETL9 protein. At the C-terminus, an extension of eight aminoacids (RSHHHHHH) are fused to the last aminoacid of the mature protein. The construct was transformed into *E. coli* strain M15 and protein overexpression and purification was done following the manufacturer’s instructions under denaturing conditions. The purity of the protein was checked by SDS-PAGE electrophoresis and Coomassie blue staining. After desalting by dialysis in 0.1 M acetic acid, the protein was lyophilised, dissolved in distilled water, and used to immunize rabbits to obtain polyclonal sera.

### *IN SITU* HYBRIDISATION AND INMUNOLOCALIZATION

A69Y maize seeds were collected at different DAP and fixed in 4% paraformaldehyde, 0.1% glutaraldehyde in 0.1 M sodium phosphate buffer pH 7.2 for 12–24 h depending on the tissue volume. Samples were dehydrated and embedded in wax (Paraplast, Sigma) using xylol as solvent. Sections 10 μm thick were affixed to glass slides treated with 3-aminopropyltriethoxylane. Sections were deparaffinised in xylol and rehydrated through an ethanol series.

For the *in situ* hybridisation, DIG- or S^35^-labeled antisense and sense probes were synthesized using T3 and T7 polymerases (Boehringer Manheim) from linearised pBluescript SK^+^ plasmids containing the *BETL9* and *BETL9like* cDNAs. Probes were partially hydrolysed with sodium carbonate. Sections were hybridized as previously described (Hueros et al., 1995), following the method of Cox and Goldberg (1988). For the detection of the hybridization signal a radiosensitive emulsion was used in the case of S^35^-labeled probes. After film developing, the signal appears as dark spots that appear bright in dark-field microscopy. Sections were counterstained with calcofluor white and photographed under UV light. In the case of DIG-labeled probes the detection was done using an anti-DIG antibody coupled with alkaline phosphatase, after addition of the enzyme substrates (Roche-diagnostics) the signal was visualized as a black–purple precipitated. Sections were counterstained with saphranine-O (Sigma).

For the immunolocalization experiments, inhibition of endogenous peroxidase was carried out by incubating the sections in 0.3% v/v H_2_O_2_ in 0.5% methanol for 10 min. Tissue was then washed in PBS and blocked with 10% normal donkey serum (Chemicon International) in PBS for 30 min at room temperature. Sections were incubated with anti-BETL9, anti-BETL9like, or preimmune sera diluted 1:200 in PBS for 2 h at room temperature. The immunoreaction was detected using horseradish peroxidase-coupled secondary antibody (Sigma) diluted 1:500 and 3′-diaminobenzidine (Sigma).

### PROMOTER ISOLATION, PLANT TRANSFORMATION, AND GUS ACTIVITY ASSAYS

Promoters of the maize *BETL9* and *BETL9like* genes were obtained from BAC genomic clones. The promoter sequences were cloned as fusions to the GUS reporter gene in *Agrobacterium*-mediated plant transformation vectors. *Z. mays* plants of the variety A188 were transformed essentially as described by Ishida et al. (1996).

Promoters of the *Arabidopsis* genes were amplified by PCR from genomic DNA using primers designed according to the information present in the *Arabidopsis* genome sequence database. 1751, 1780, 1013, and 1001 bp of sequence upstream of the coding region start codon were amplified from the At1g32280, At4g30880, At4g33550, and At5g56480 genes, respectively. To prepare the *Arabidopsis* reporter lines, these promoter sequences were ligated upstream of the GUS reporter gene in the pBI101.3 plasmid (Clontech). *A. thaliana* Col-0 plants were transformed following the floral dip method as described by Clough and Bent (1998).

Expression of the GUS gene was detected by histochemical staining according to the method of Jefferson et al. (1987). Tissues were stained for GUS in a medium containing 0.5 mg/ml X-glucuronide (Duchefa), 0.5 mM K^+^-ferrocyanide, 0.5 mM K^+^-ferricyanide, 10 mM Na_2_EDTA, 50 mM phosphate buffer, pH 7, 0.1% Triton X-100, and 20% methanol.

## Conflict of Interest Statement

The authors declare that the research was conducted in the absence of any commercial or financial relationships that could be construed as a potential conflict of interest.
